# Simulating the spatiotemporal variations of oasis rural settlements in the upper reaches of rivers of arid regions in Xinjiang, China

**DOI:** 10.1371/journal.pone.0275241

**Published:** 2022-09-29

**Authors:** Ling Xie, Hongwei Wang, Suhong Liu

**Affiliations:** 1 College of Resources and Environmental Sciences, Xinjiang University, Urumqi, China; 2 School of Environment and Resources, Guangxi Normal University, Guilin, China; 3 Faculty of Geographical Science, Beijing Normal University, Beijing, China; Gebze Teknik Universitesi, TURKEY

## Abstract

Rural settlements in oasis are primary habitations, and their changes are related to natural environment and anthropogenic activities. The spatiotemporal variations of rural settlements in an oasis are significant in arid regions. In this study, Qipan Township (QPT) and Yamansu Township (YMST) were chosen as a case study and validation case, respectively. Datasets, including Landsat images in 2002, 2010, and 2018, were collected. The cellular automata (CA)-agent-based model (ABM) and patch-generating land use simulation (PLUS) model were used to simulate the spatiotemporal dynamic variations of rural settlement and other land use types in the oasis in this study. Natural environmental, socioeconomic conditions, and human decision-making are the three driving factors that were used in the model. Human decision-making involves the actions of two types of agents: authority agent and resident agent. On the basis of land use data of 2002 and 2010, the rural settlement and other land use in 2018 were predicted using the CA-MAS and PLUS models. The following results were obtained: First, human decision-making behaviors were the leading factor in the changes of rural settlements in the CA-ABM model. Second, CA based on multiple random seed (CARS) of PLUS could better simulate the spatiotemporal variations of QPT rural settlements than CA-ABM and linear regression of PLUS. Similarly, CARS of PLUS also simulated the spatiotemporal evolution of rural settlements in YMST with high accuracy. Third, the areas of croplands, roads, and residential lands in QPT will expand to 20.7, 5.7, and 4.6 km^2^, respectively, in 2026, but the unused land will shrink, as predicted by CARS of PLUS. This study provides a scientific basis for the environmental protection of rural settlements in the oasis and sustainable settlement planning in arid regions.

## Introduction

The evolution of rural settlements in an arid-region oasis is the outcome of interactions between humans and the natural environment, as well as the long-term development of human societies. Settlements are the basic units of rural areas [1]. Rural settlements in an oasis are the geographical space carrier of agricultural production and rural ecosystems [2,3]. According to reports, 60% of the population in Xinjiang still lived in rural settlements in oasis in 2017 (Xinjiang Statistical Yearbook, 2018). Even though the oasis encompasses only a small part of the region, it produces more than 90% of the social wealth and supports more than 95% of the population in Tarim Basin [4]. In Tarim Basin, small oasis rural settlements are distributed along the upper reaches of the river, and those rivers originate from the mountains around Tarim Basin. The development and migration of small oases in the upper reaches of the river seriously affect the oases in the lower reaches of the river in Tarim Basin and the environment of Tarim Basin. The study on the spatiotemporal evolution of rural oasis settlements in the upper reaches of Tarim Basin is the basis for further research on the impact of the spatiotemporal evolution of rural oasis settlements on the natural environment in Tarim Basin. Therefore, studying the spatiotemporal changes and driving factors of rural oasis settlements in the upper reaches of river in Tarim Basin is of great significance. The evolution of rural settlements in an oasis has an important impact on the ecosystem of arid areas [5]. However, the land use change in an oasis has been controversial for a long time due to its complex process, dynamics, and driving force [6,7]. The dynamics of rural settlements in the oasis of the arid region have long been neglected [8], and the spatiotemporal variations of rural settlements and land use were seldom explored [9,10]. Similarly, fewer studies have been conducted on oasis rural settlements in the upper reaches of Tarim Basin.

Studies on the spatial and temporal evolution of settlements have mostly focused on identifying rural settlements to accurately reflect their spatial and temporal changes [2,9,11,12], exploring the drivers of rural settlement transformation at the grid scale, which are often ascribe to socioeconomic factors and the natural environment level, without considering the impact of the decision-making behavior of managers and residents [13] or reflecting the changes in the landscape pattern of rural areas only at the level of residents’ decisions [14]. Various methods have been used in dynamic modeling and geographic simulation of land use changes. For example, mathematical models based on spatial characteristics, such as the logistic regression and cellular automata (CA) model [15–18], and CA and Markov model [19–21], are widely used to simulate land use changes. Nevertheless, these models are not applicable for exploring the impact of the interaction between managers and residents on simulation results. The agent-based model (ABM) considers the comprehensive effects of both nature and humanity on the simulation results of land use change processes based on the complex adaptive system (CAS). In many studies [22–27], ABM was adopted to conduct computerized simulation, and results show that the appropriate introduction of human decision-making behaviors in agent-based geographic simulation models is important [14,28]. In addition, hybrid geo-computation models such as CA-ABM [29–31] and artificial neural network-ABM [24,32] were developed to support the exploration of land use changes, especially the dynamic simulation of rural settlements. Hybrid models could simulate the dispersive individual decision-making behaviors, capture the complex spatial interactions among individual decision-making behaviors [33], and represent the real process of land use change. Meanwhile, a new hybrid model called patch-generating land use simulation (PLUS) was developed and is widely used. The PLUS model integrates a rule-mining framework based on the land expansion analysis strategy (LEAS), and a CA model based on multitype random seeds (CARS) and can illustrate the drivers of land expansion and project landscape dynamics. In our research, we took the agent result as the driving factor and implanted it into PLUS. LEAS can help researchers investigate underlying transition rules. It combines simulation with knowledge discovery and policy-making processes and can provide vital information for multiple users (e.g., researchers, planners, and policymakers) [35]. However, the CA-ABM and PLUS hybrid modeling technology was mainly used to predict urban or metropolitan expansion at different scales. We still found the application of relevant research methods in the spatiotemporal changes of rural oasis settlements [34–36]. The coupling effect between the natural environment and human behaviors in oasis rural settlement evolution remains poorly understood to date. In this study, CA-ABM and PLUS models were used to simulate rural oasis settlement evolution and land use change in the upper reaches of Tarim Basin. The rural settlements and land use change of oasis in the upper reaches of Tarim Basin are the basis of the next research on environmental impact assessment of the whole Tarim Basin.

The spatiotemporal evolution of rural settlements in the upper reaches of the oasis river in the Tarim Basin has long been neglected, which is why the mechanism of driving factors for the spatiotemporal evolution of these settlements is unclear. This issue is of great significance to the sustainable development of Tarim Basin and even for Xinjiang. The evolution process and mechanism of rural settlement pattern in the upper reaches of Tarim Basin is complex, which also has a profound impact on the natural environment of Tarim Basin. Therefore, more in-depth case studies and comprehensive studies are needed on oasis settlements and other types of land use in Tarim Basin. Since the beginning of the 21st century, the evolution of rural settlements and their land use patterns in the upper reaches of the Tarim river has entered a critical stage. Exploring the evolution mechanism based on the long-term changes in the land use pattern of oasis settlements in the upper reaches of the river can provide a reference for measuring the impact of human activities in the area on the natural environment of the Tarim Basin. In this study, the CA-ABM model combines CA with human decision-making behaviors (including individual decision-making behavior and government decision-making behavior considering policy planning) to simulate the evolution of rural settlements in the upper reaches of the oasis river. The PLUS model integrates the random seed mechanism of human decision-making behaviors based on CA and random forest to simulate the evolution of rural settlements in the upper reaches of the oasis river. Two models were introduced to determine the direction of rural settlement change in the oasis. The study aims to find a suitable model to simulate the dynamic spatiotemporal variations of rural settlements in Tarim Basin to explore a set of applicable spatial and temporal evolution model coefficients of rural settlements in the upper reaches of oasis rivers in Tarim Basin. This study also aims to provide reference suggestions for the planning of oasis settlements in Tarim Basin and the sustainable development of small oases in the upper reaches of oasis rivers in the area. In addition, this study reveals the inherent mechanism and driving factors of the evolution and presents the variations of other land use types with the conversion of rural settlements in the oasis to further study the impact of rural settlements and land use change on the natural environment of Tarim Basin.

## Materials and methods

### Study area

**Fig 1:**
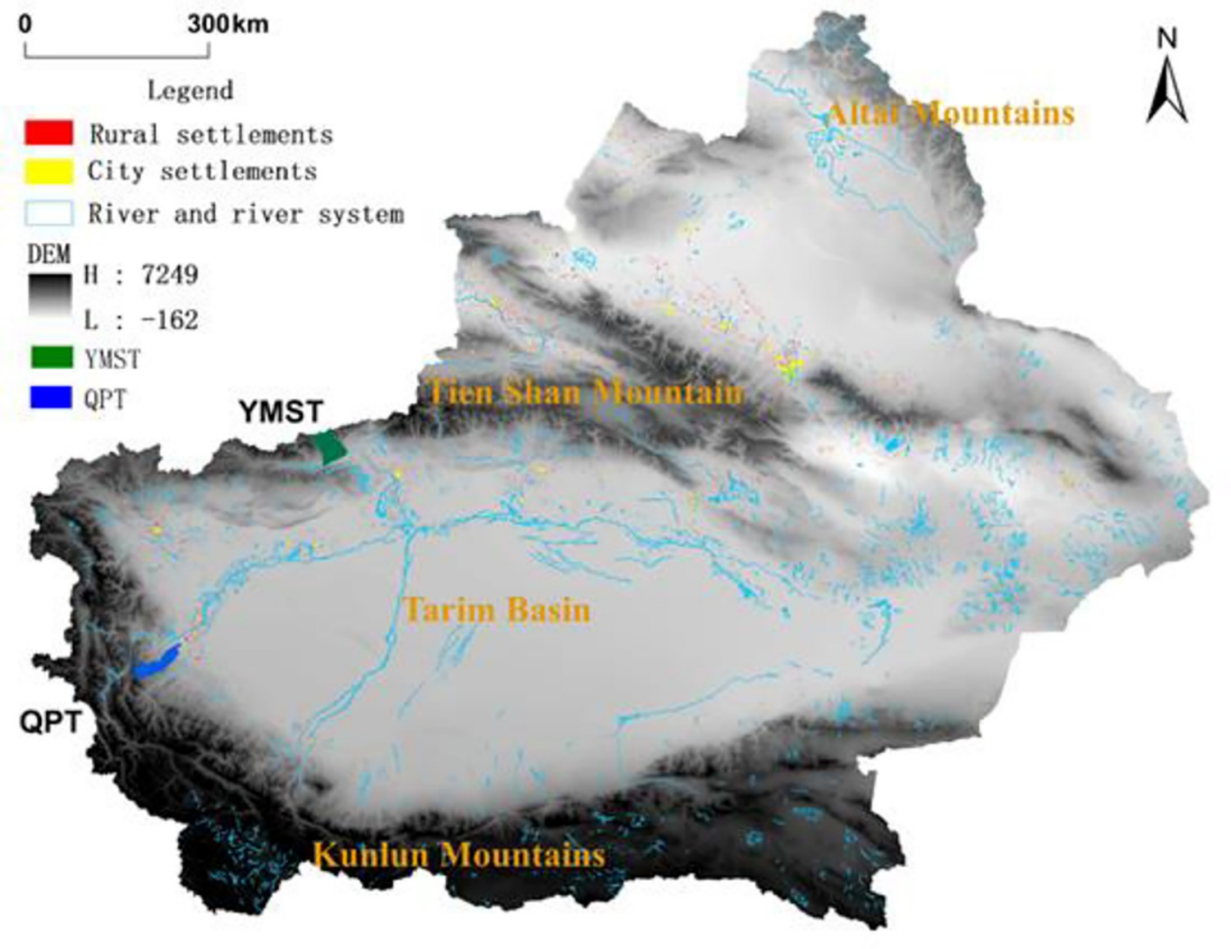
Geographic location of the study area. (The original map was downloaded from The Gateway to Astronaut Photography of Earth Website (https://eol.jsc.nasa.gov/SearchPhotos/). The map downloaded from this website is free and open to scholars; thus, we do not need to supply copyright permission).

Qipan Township (QPT) (37.309°N–37.978°N, 76.349°E–77.098°E) and Yamansu Township (YMST) (41.013°N–41.558°N, 79.181°E–78.391°E) are located in the southwest and northwest margin of Tarim Basin in Xinjiang, China (Fig 1). They border Kunlun Mountains in the south and Tianshan Mountains in the north, respectively. The Yarkand River Basin Oasis is in the northeast of QPT, and the Aksu River Basin Oasis is in the southeast of YMST. They are all small oasis rural settlements along the upper reaches of the river of the Tarim Basin. Many similar rural oasis settlements are present around the Tarim Basin, and we selected them as representatives. The local climate is characterized by an arid continental climate, annual average temperature of 10°C, and annual average precipitation of 100.3 mm, and is a combination of mountain-oasis-desert eco-geomorphic landscapes [37]. Landscapes in more than 95% of the study area are mountains and Gobi deserts. QPT has 13 villages, with a population of 16323, and YMST has 7 villages, with a population of 8956 in 2015. The economic income in QPT and YMST mainly relies on plantation, forestry, fruits, and animal husbandry. Most oasis rural settlements in the upper reaches of the Tarim Basin are similar to QPT and YMST.

### Data sources

**Fig 2:**
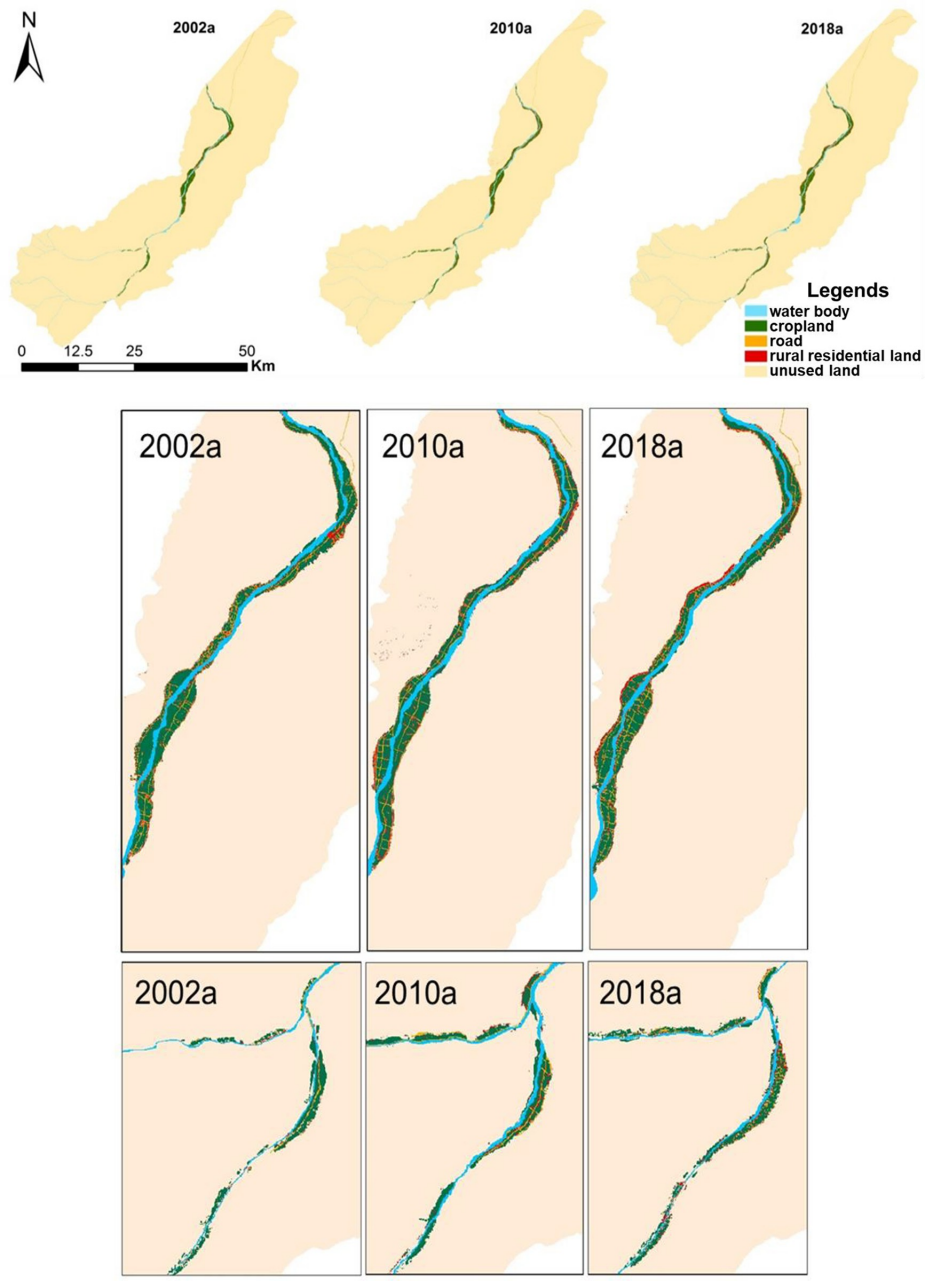
Classification map of land uses in the study area. (Categorical data are from the Landsat Thematic Mapper images of the United States Geological Survey [https://earthexplorer.usgs.gov] [Path/Row number: #148 #34; Date: 2002/06/22; 2010/08/23; 2018/05/09]. Validation data are from the MSS&PAN images of the GF-1 Satellite in 2018. Those data products are at level 1A and cloud coverage was less than 10%. Furthermore, 100 training samples were selected from each class by using support vector machine. The separability of the sample is more than 1.9, and the overall classification accuracy reaches 80%, The classification results of GF-1 are consistent with those of LANDSAT in 2018).

The data used in this study are listed in Table 1. The landscapes in this study area were divided into five types (Fig 2): cropland (including woodland, grassland, and garden within the settlements of the study area), water body (including river and reservoir), roads, rural residential land, and unused land (including Gobi Desert and mountains).

**Table 1:** Summary table of data source information.

Data	Types	Dates	Resolutions	Sources
LANDSAT	TM image	2002/2010/2018	30 m	USGS
GF-1	TIF image	2018	2m/8m	Geospatial Data Cloud
Other Digitalized Data	Vectors		The data of hospitals, schools, administrative centers and ecological reserves	Yecheng Land Resources Bureau Wushi Land Resources Bureau
Socio-economic data		2002/2010/2018		Statistical Bulletin of National Economy, Yecheng/ Wushi Yearbook

## Methods

### Methodological framework

**Fig 3:**
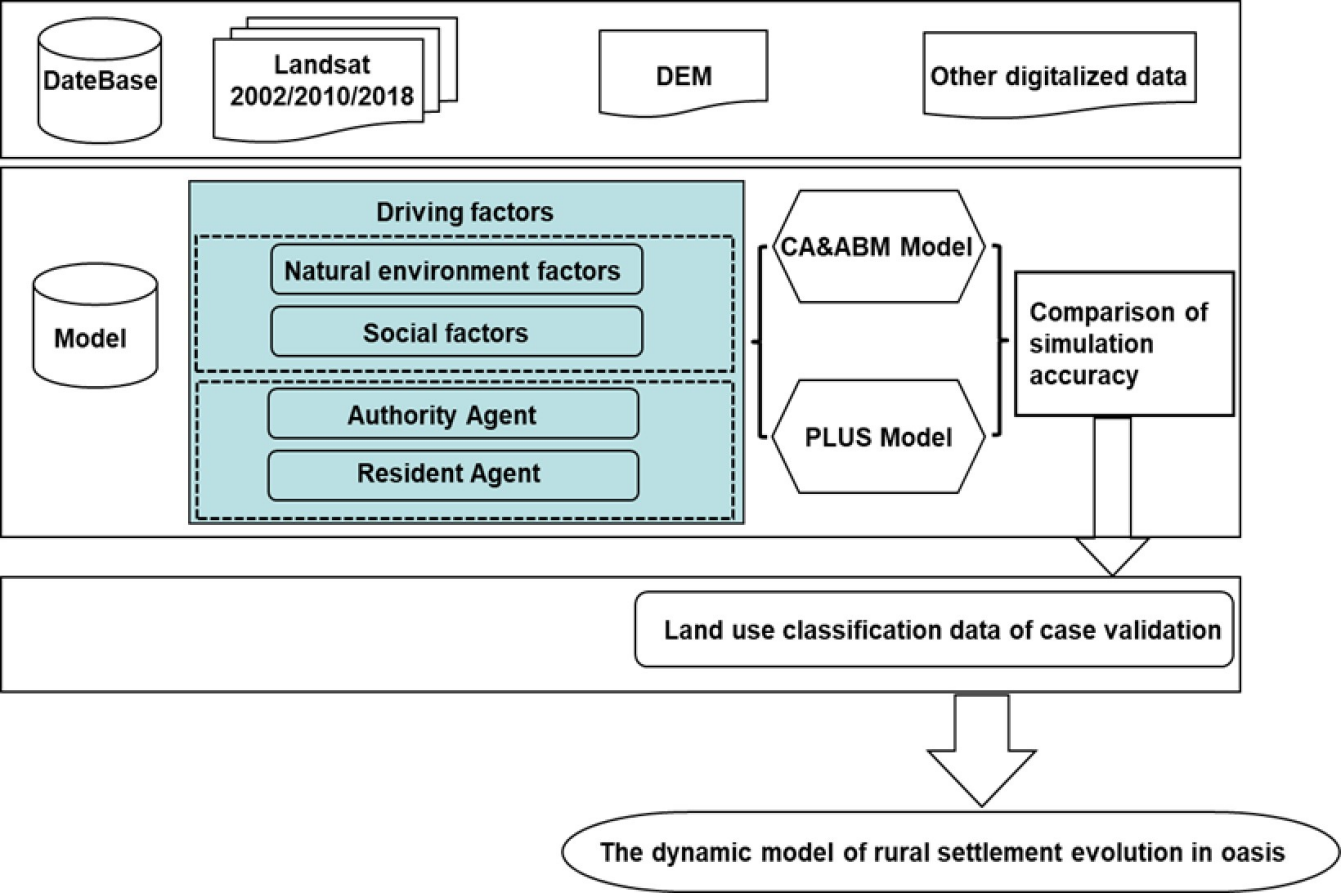
Conceptual framework of the changes of rural settlements in oasis based on CA and ABM.

The simulation framework of rural settlements in the study area is shown in Fig 3.

**Fig 4:**
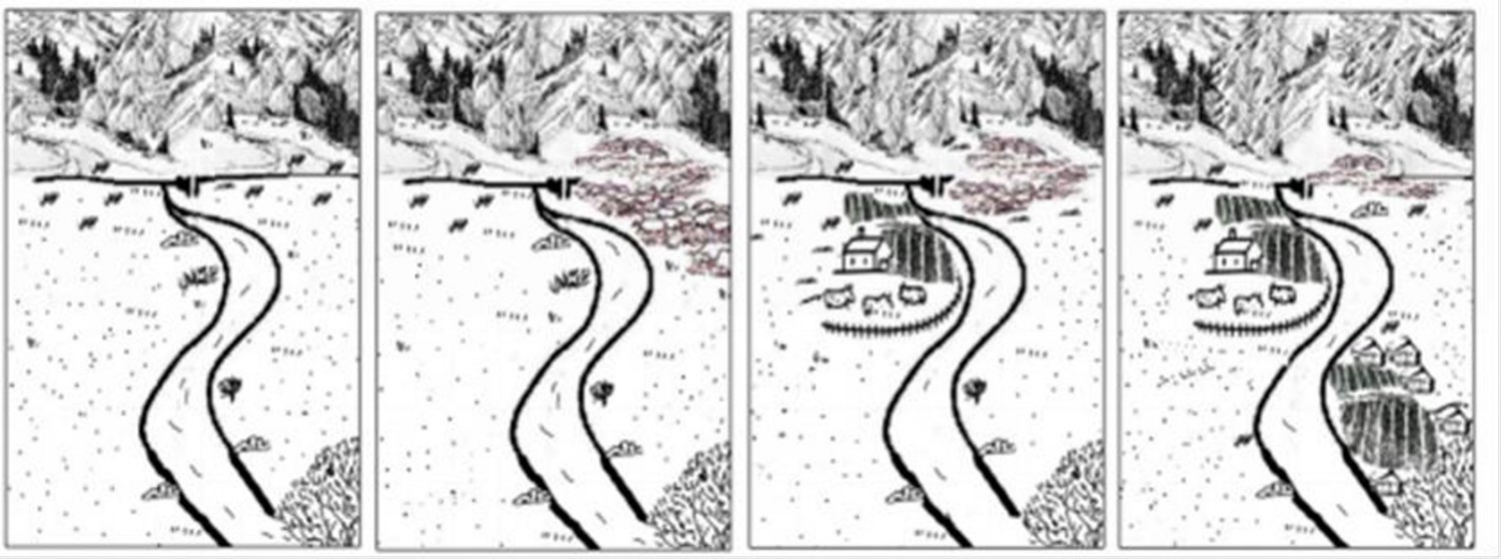
Change patterns of rural settlements in the study area.

In the study, the CA-ABM and PLUS models were employed to depict the evolution of rural settlements. Fig 4 depicts the evolution process of oasis rural settlements in the upper reaches of Tarim Basin.

### CA-ABM models

#### Cellular automata model

The land grid cell of the landscape system in this study has similar characteristics with the cell of the CA. Hence, the CA is applicable to simulate the land use change in this landscape system. When each land grid in the landscape system is viewed as a cell of CA, the spatiotemporal variations of land use can be derived as follows (Eq 1) [24]:

S(t+1)=f(St,Nt), (1)

where St is the state of a cell at time t; Nt is the state of the cell in the neighborhood at time t; f is the transition rule determined by the transition probability; and S(t+1) is the state of the cell at time (t + 1).

In this study, the cells are the land use grids, and each lattice has 8 neighboring cells. The time step is 9 years, and the transition rule adopts a 3 × 3 neighborhood. The cell state in the landscape system was not fixed, and the transition of cells to rural settlements was determined by different driving factors [38,39]. CA’s transition rules use a 3 × 3 neighborhood to judge the land use type in the future. The state of each cell is affected by the states of its 8 neighboring cells in the filter. Correspondingly, the future land use of a cell is determined by the land use types in its 8 neighboring cells.

#### Agent-based model

ABM is widely used to explore the influences of micro-level individual decision-making behaviors and macro-level decision-making behaviors on simulation results. CA model has a spatial self-organization ability. Therefore, ABM combined with CA can be used to simulate the decision-making behaviors of micro-agent in a complex space [31,40] and analyze complex geographical phenomena [22,41]. Spatiotemporal variations of settlements were explored in this work by using CA from the perspective of the landscape patch of land use, and the evolution of rural settlements under the coupling effect of the authority and residents was explored with ABM from the perspective of human behaviors.

### PLUS model

PLUS was developed purely in C++ language. The parallel technology of PLUS software is from the High-performance Spatial Computational Intelligence Lab @ China University of Geosciences (Wuhan) (https://github.com/HPSCIL) [42]. It is a land use simulation model coupled with Markov chain and random forest algorithm. The conversion rule mining strategy and landscape dynamic change simulation strategy are improved compared with the previous land use simulation models. The model has the following advantages. First, a new analysis strategy can better derive the incentives of various types of land use changes. Second, it contains a new multi-type seed growth mechanism, which can better simulate multiple patch-level changes in similar land uses. Third, with multi-objective optimization algorithms, the simulation results can better support planning policies to achieve sustainable development.

With random seed generation and threshold decreasing mechanism, the PLUS model can dynamically simulate the automatic generation of patches in time and space under the constraint of development probability. When the neighborhood’s effect of land use *k* is 0, the probability surface ($P_{i,k}^{1}$) for each land-use type is determined below (Eq 2)

$$OP_{i,k}^{1,t}=\{\begin{array}{l} P_{i,k}^{1}\times (r\times \mu_{k})\times D_{k}^{t},if\Omega_{i,k}^{t}=0,and,r<P_{i,k}^{1}\\ P_{i,k}^{1}\times \Omega_{i,k}^{t}\times D_{k}^{t} \end{array},$$ (2)

where *r* is a random value ranging from 0 to 1, and μ_k_ is the threshold for producing new land use patches. To generate new land use patches, the land use type *k* is used. Seeds can generate new types of land use and expand into a series of new patches. To control the generation of multiple patches, the threshold declining rule is proposed for the competition process. If the new land use type wins in a round, then a declining threshold τ is used to determine the candidate land‐use type *c* as follows (3 and 4):

$$IF\sum_{k=1}^{N}\left|G_{c}^{t-1}\right|-\sum_{k=1}^{N}\left|G_{c}^{t}\right|<step,Then,d=d+1,$$ (3)

$$\left\{ \begin{array}{l} Change,P_{i,c}^{1}>\tau ,and,TM_{k,c}=1\\ Unchange,P_{i,c}^{1}\leq \tau ,or,TM_{k,c}=0 \end{array}\;\tau =\delta^{d}\times r,\right.$$ (4)

where "Step" is the step size used in the simulation of the land use; δ is the factor of attenuation; *r* is a stochastic value distributed with a mean of 1; *d* is the number of attenuation steps; and TM_k,c_ is the transformation matrix for deciding when type *k* is permitted to be converted into type c of land use. The driving factors of the PLUS model are shown in Table 1. The specific variables of the PLUS model were set as follows: the number of regression trees is 20, the sampling rate is 0.01, and mTry is 10. These parameter settings were obtained from the model manuals.

### Driving factors

#### Land use layer

Land use is the core of simulating the spatiotemporal variations of rural settlements. In this study, we divided the land into rural residential land and non-rural residential land, and then assigned the values of 1 and 0 to rural residential land and non-rural residential land, respectively.

#### Natural environmental layer

The natural environment has much larger effects on changes in rural land use, especially the environment constrained by water resources in arid regions. In this layer, the digital elevation model (https://srtm.csi.cgiar.org/), slope, aspect, and distances to rivers, cropland and unused land were considered the influencing factors of rural residential land change.

#### Socioeconomic layer

Social and economic conditions refer to the status in which rural residents can conveniently use public infrastructure services and agricultural production activities. Thus, distance to public facilities such as schools, hospitals, roads, and administrative centers is the important factor that affects residents’ decision. In the evaluation of the utility of public facilities, distance decay functions were used to reflect the available space.

Therefore, logistic regression analysis was performed to determine the probability that a cell containing a certain land use type would be converted into another land use type [15]. The transition probability of a land use grid cell influenced by different factors can be calculated. The logistic regression equation is provided as follows (Eqs 5 and 6) [43]:

$$\log(\frac{P_{CA}}{1-P_{CA}})=\beta_{0}+\beta_{1}X_{1}+\beta_{2}X_{2}+\cdots +\beta_{n}X_{n},$$ (5)

$$P_{CA}=\frac{1}{1+\exp[-(\beta_{0}+\beta_{1}X_{1}+\beta_{2}X_{2}+\cdots +\beta_{n}X_{n})]},$$ (6)

where *Pca* is the transition probability of a cell transited from other land use types to rural settlements in the oasis and can be calculated with raster calculator in ArcGIS10.2; X_1_, X_2_, X_3_, and X_n_ are driving factors; β_1_, β_2_, β_3_…βn are regression coefficients obtained by the binary logistic regression tool in the SPSS22.0 software; and β_0_ is a constant. Eighty-seven random points within QPT were extracted to calculate the random values of driving factors. The random points were generated by the ArcGIS "Create Random Point" tool.

The Euclidean distance from each cell to all driving factors was obtained with ArcGIS, and then the distance in different element layers was normalized as in Eq 7 to eliminate the effects of dimensions and variations

$$S{'}_{\left(ij\right)}=\left\{ \begin{array}{l} 1\\ \frac{S_{\left(ij\right)}-MinS_{\left(ij\right)}}{MaxS_{\left(ij\right)}-MinS_{\left(ij\right)}} \end{array}\right.\;\begin{array}{l} MaxS_{\left(ij\right)}=MinS_{\left(ij\right)}\\ MaxS_{\left(ij\right)}\neq MinS_{\left(ij\right)} \end{array},$$ (7)

where S_ij_ denotes the Euclidean distance from each cell to the driving factors; MaxS_ij_ is the maximum distance; MinS_ij_ is the minimum distance; and S’_(ij)_ is the normalized data.

#### Human decision-making layer

ABM can be used to simulate the interactions between macro decision-making behaviors and its micro behavioral agents in a certain situation [26]. In this study, the behavioral agents are the decision-making behaviors of the resident agent and the governmental agent. To explore the decision-making behaviors of residents, a random sampling survey was conducted among residents. The survey results indicated that the top four factors in decision-making behaviors were "Kindred," "Distance to road," "Distance to school," and "Distance to cropland."

The household decision-making behaviors of residents were simulated with the minimal cumulative resistance (MCR) model [44]. We improved the MCR model based on the consideration of the changes of rural settlements. The improved MCR model was formulated similar to the friction equation as follows (Eq 8):

$$F=\mu F_{n},$$ (8)

where *μ* is the coefficient of friction, and *Fn* is the positive pressure (Eq 9)

$$F_{\left(n\right)}=f_{R}{}_{\left(n\right)}*S_{R(n)}{}^{-1}(f_{R}{}_{\left(n\right)}=0.1,0.3,0.5,0.7,0.9),$$ (9)

where *f*_*R*(*n*)_ is the coefficient of friction in different buffer areas (set to 0.1, 0.3, 0.5, 0.7, and 0.9); $S_{R(n)}{}^{-1}$ is the reciprocal of the total area of rural residential land in the study area; and *f*_*R*(*n*)_ is the resistance to the conversion of settlements. In this study, the total area of the residential land in different buffer areas was viewed as the positive pressure, and the radius of the buffer area was determined by the results of the questionnaire. The conversion resistances of rural settlements in different buffer areas under the influences of kindred, roads, schools, and cropland are respectively expressed as follows (Eqs 10–13):

$$F_{Kindredsum}=\sum_{i=0.1}^{5}\left(F_{R(0.1)}+F_{R(0.3)}+\cdots +F_{R(0.9)}\right);$$ (10)

$$F_{Roadsum}=\sum_{i=0.1}^{5}\left(F_{R(0.1)}+F_{R(0.3)}+\cdots +F_{R(0.9)}\right);$$ (11)

$$F_{Schoolsum}=\sum_{i=0.1}^{5}\left(F_{R(0.1)}+F_{R(0.3)}+\cdots +F_{R(0.9)}\right);$$ (12)

$$F_{Croplandsum}=\sum_{i=0.1}^{5}\left(F_{R(0.1)}+F_{R(0.3)}+\cdots +F_{R(0.9)}\right).$$ (13)

Therefore, a dynamic stochastic model based on the household decision-making behaviors of residents is established with the range attenuation function as follows (Eq 14):

$$P_{1}=\frac{1}{F_{R(n)}}(F_{R(n)}=F_{Kindredsum}+F_{Roadsum}+F_{Schoolsum}+F_{Croplandsum}).$$ (14)

The household decision-making behaviors under the governmental regulation are influenced by village committee and local township government, and the dynamic stochastic model based on the government decision-making behaviors is established as follows (Eq 15):

$$P_{2}^{'}=\alpha *P_{tg}+\beta *P_{vc}+\varepsilon ,$$ (15)

where *P*_*tg*_ and *P*_*vc*_ are the transition probabilities of rural settlements in a homogeneous space affected by the township government and the village committee, respectively; d_tg_ and d_vc_ are the distances to the township government and the village committee, respectively; *ε* is a random interference coefficient obtained from the equation: *ε* = [−ln(*rand*)]^∂^ [45]; and *α* and *β* are the coefficients of *P*_*tg*_ and *P*_*vc*_ can be obtained through the analytic hierarchy process [46].

The range attenuation function was also employed here. To guarantee the significance of the fraction and avoid the zero denominators, the mathematical constant *e* was used to express the attenuation relationship between the distance and the probability (Eqs 16 and 17)

$$P_{tg}=e^{\left(-d_{tg}\right)},$$ (16)

$$P_{vc}=e^{\left(-d_{vc}\right)}.$$ (17)

The changes of rural settlements in the study area are constrained by land planning, such as the planning of ecological reserves, roads, and rivers. Thus, the transition probability of the rigid constraint area is 0, and the probability of the non-rigid constraint area is 1. The constraint function is expressed as follows (Eq 18):

$$CON_{ij}\{\begin{array}{l} P=0(\mathrm{rigid}\;\mathrm{constraint}\;\mathrm{area})\\ P=1(non-\mathrm{rigid}\;\mathrm{constraint}\;\mathrm{area}) \end{array}.$$ (18)

Hence, the transition probability of rural settlements in the study area under the influence of macro-level planning is expressed as follows (Eq 19):

$$P_{2}=P_{2}^{'}\times CON_{ij}.$$ (19)

The transition direction and the scale of rural settlements in the study area are determined by the decision-making behaviors of both the residents and the government. Therefore, under the influence of subjective human behaviors, the transition probability of the land use based on the ABM can be expressed as follows (Eq 20):

$$P_{ABM}=\delta *P_{1}+\theta *P_{2},$$ (20)

where *P*_*ABM*_ refers to the transition probability of the ABM; *P*_1_ is the transition probability under the influence of individual decision-making behaviors; *P*_2_ is the transition probability under the influence of government decision-making behaviors of macro-level planning; *δ* is the influencing coefficient of *P*_1_; *θ* is the influencing coefficient of *P*_2_; and *δ*+*θ* = 1 in this study. A multi-scenario simulation was used to accurately simulate the changes of rural settlements. Moreover, the selected factors are the important driving forces of the changes of rural settlements, which are strongly constrained by water resources in the study area. Thus, the CA-ABM model developed for the spatiotemporal variations of rural settlements in the upper reaches of Tarim Basin in this paper is expressed as (Eq 21)

$$P=\lambda *P_{CA}+(1-\lambda )*[\delta *P_{1}+\theta *P_{2}].$$ (21)

On the basis of the established CA-ABM model, interactive data language (IDL) was employed to write a program to simulate the future changes of rural settlements in an oasis. Through multi-scenario simulation, the transition probability was set. If the probability exceeds 0.5, then the land would be changed into rural settlements; otherwise, it would be viewed as its original land use.

## Results

### Effect analysis of the driving factors of rural settlement evolution in oasis on landscape patch

**Fig 5:**
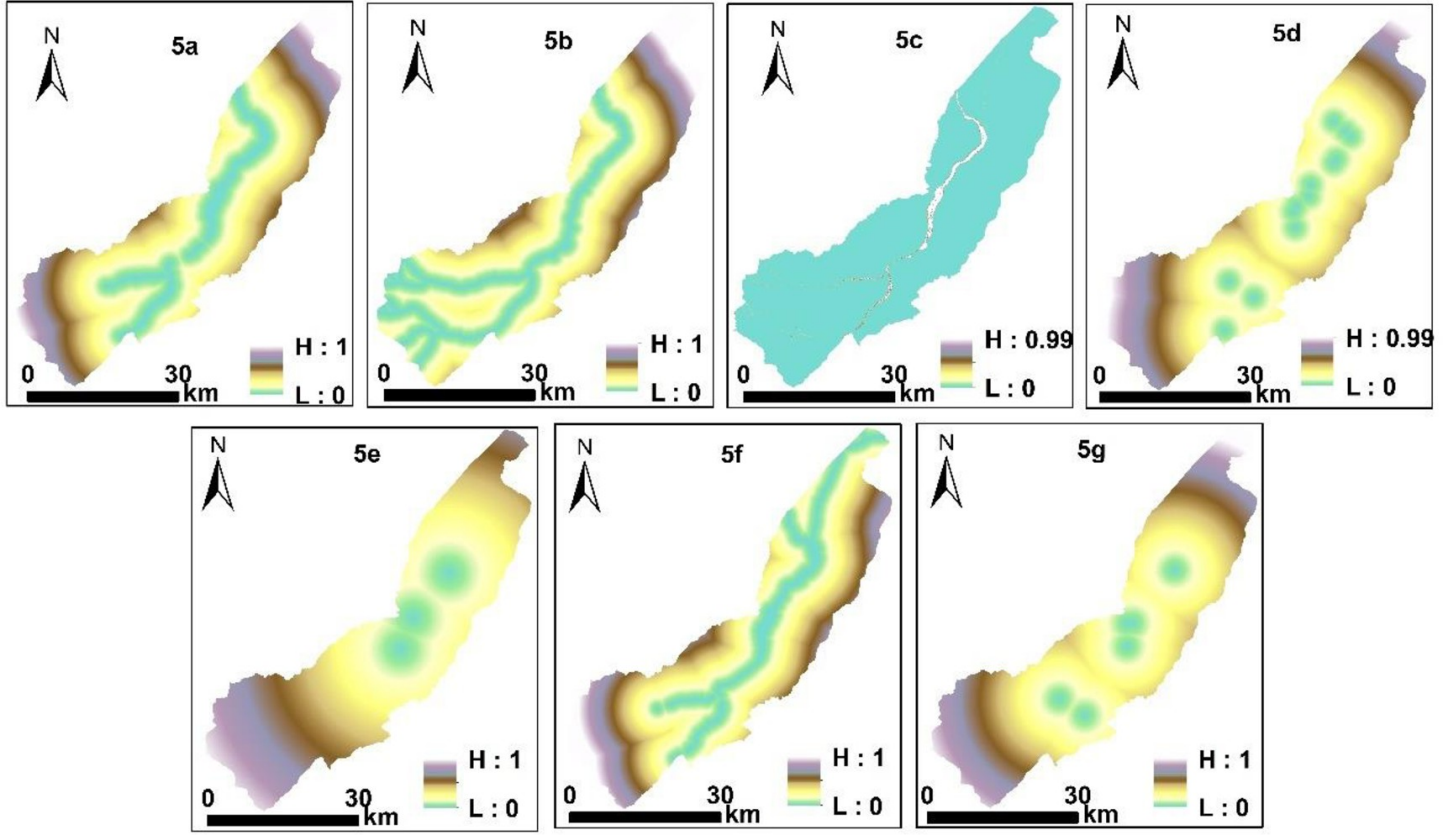
Spatial expression of the influences of the driving factors (Software: ArcMap 10.2.0, http://www.esri.com. Figures were generated with ESRI using author-owned data). 5a Transfer probability of cropland; 5b. Transfer probability of water body; 5c. Transfer probability of unused land; 5d. Transfer probability of administrative centers; 5e. Transfer probability of hospital; 5f. Transfer probability of road; 5g. Transfer probability of school).

The β values in the binary logistic analysis are shown in Table 2. Except for the factor of "distance to unused land" having a positive correlation (β = 12.731), other variables had a negative correlation with the evolution of rural settlements in oasis. Fig 5 shows the transition probability of rural settlements under the influence of each driving factor in the landscape patch layer in the study area. In the landscape patch layer, the comprehensive probability of rural settlements under the influences of all driving factors in the study area ranges from 0.001 to 1. Multiple driving factors were selected, which is why the transition direction was dispersed on the landscape patch layer.

**Table 2:** Coefficients of the driving factors in the logistic regression analysis.

	Factors	β values	Variables
Natural environmental factors	DEM	-1.732	X_1_
	Slope	-1.203	X_2_
	Aspect	-0.078	X_3_
	Distance to cropland	-3.055	X_4_
	Distance to river	-1.467	X_5_
	Distance to unused land	12.731	X_6_
Social-economic factors	Distance to school	-.2.250	X_7_
	Distance to hospital	-1.693	X_8_
	Distance to road	-0.457	X_9_
	Distance to administrative centers	-1.279	X_10_
	Constant	-3.862	

### Land use conversion probability of rural settlements based on CA-ABM

According to Formula (6) and the binary regression coefficients of all influencing factors in Table 2, the probability of other land types being converted into rural settlements under binary regression is obtained, and the conversion probability range is 0.0015–1.

**Fig 6:**
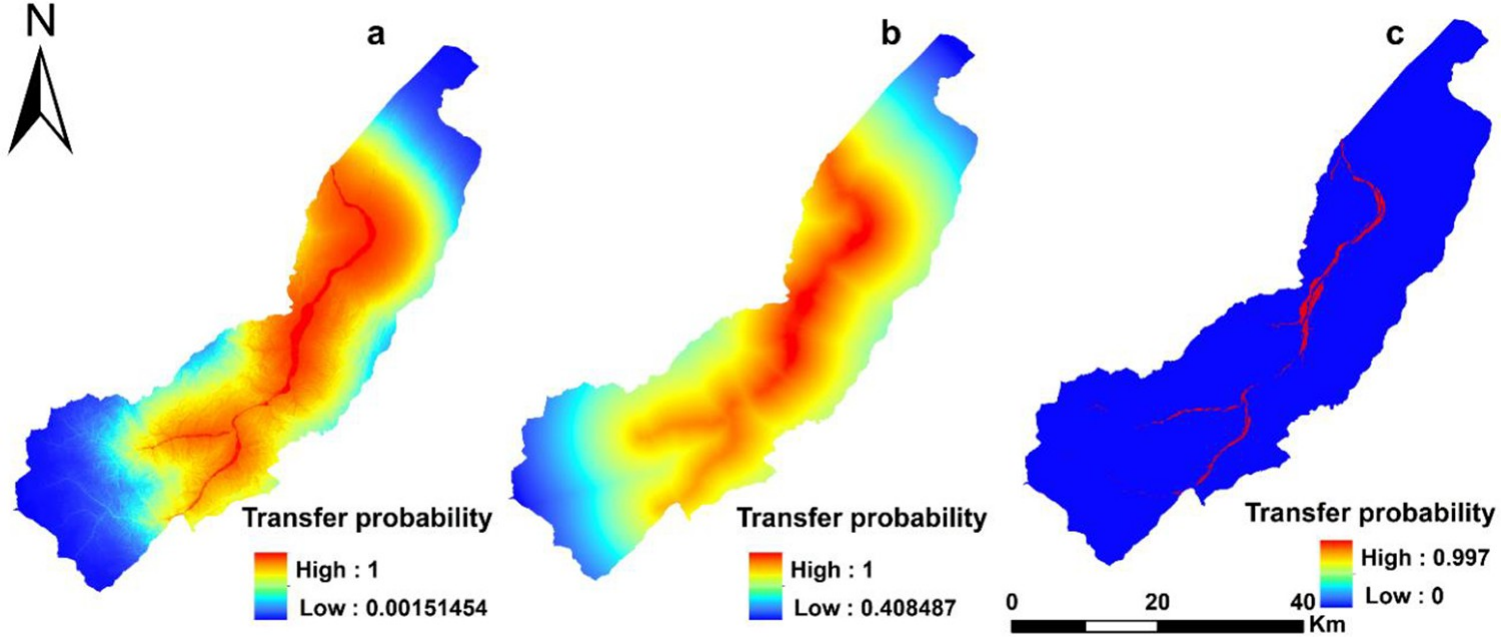
Transition probability of rural settlements in QPT. (Software: ArcMap 10.2.0, http://www.esri.com. Figures were generated with ESRI using author-owned data).

The residents are the micro behavioral agents. On the basis of the improved MCR model, the comprehensive transition probability of rural settlements under the influences of kindred, the convenience of transportation, education, and agricultural production is shown in Fig 6A. The probability was high and ranged from 0.408 to 1. The higher probability indicated less conversion resistance of other land use types into rural residential land under the influences of local residents’ wishes.

The authorities are the macro decision-making agents. Fig 6B shows that the transition probability of rural settlements in the study area under the influence of governmental regulation ranges from 0 to 0.997. The warm-colored areas in Fig 6B are the areas with a high transition probability close to 1. Usually, administrative areas have better infrastructure, such as better transportation conditions and more educational and medical resources. Thus, rural settlements are built in administrative areas. Vice versa, the blue areas in Fig 6B are restrained by ecological reserve plans or the general plan involving ecological reserves (the natural forest, snow, and permanent snow area in the north of Kunlun Mountains), prime cropland preservation area, special land for scenic spots, roads, rivers, reservoirs, sandy land without vegetation, and other rigid constraint areas. The transition probability in these areas is 0.

### Land use conversion probability of rural settlements based on the PLUS

**Fig 7:**
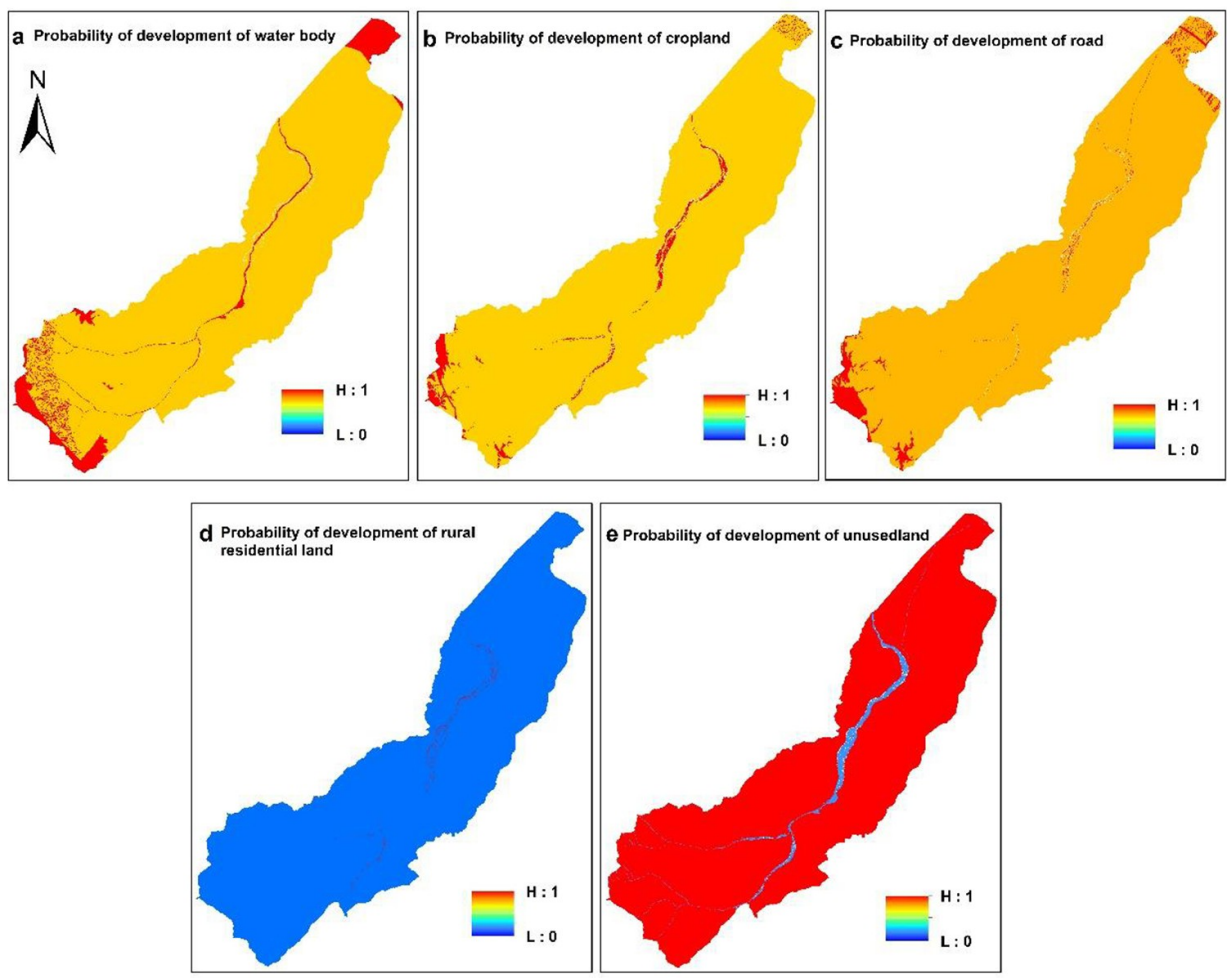
Development probability of land in QPT. (Software: ArcMap 10.2.0, http://www.esri.com).

With the LEAS module of the PLUS model, we extracted the parts of land expansion between the two periods of land use change based on the land use data in 2010 and 2018 of QPT. The random forest algorithm was used to excavate the driving factors of land use expansion one by one. The development probability of various types of land use and the contribution of driving factors was obtained for the expansion of various types of land use in this period (Fig 7). The driving factors were consistent with those obtained with the CA-ABM model. With random seed generation and threshold decreasing mechanism, the PLUS model can dynamically simulate the automatic generation of patches in time and space under the constraint of development probability. Fig 7A shows the development probability of water land. The area of water bodies in the south of Kunlun Mountains has a higher probability of land development because the Qipan River originates from the northern foot of Kunlun Mountains. Fig 7B shows the development probability of cropland. The cropland in arid regions is closely related to water resources [47] and the distribution of water body plays a decisive role in cropland development probability. The development probability of road land is close to that of water body and cropland (Fig 7C). The development probability of rural residential land is close to that of the original rural residential land. All unused lands are converted to rural settlements with the same probability (Fig 7D). The development probability of unused land is much lower than that of other land (Fig 7E).

### Comparison of the accuracy of multi-model simulation results

On the basis of the CA-ABM model, we established the dynamic model of spatiotemporal variations of rural settlements in QPT. According to the land use classification of the study area in 2010, IDL programming language was used to realize multi-scenario simulation. We simulated the evolution of rural settlements and the land use change in the study area in 2018 and verified the accuracy with the land use classification results in 2018. When *δ* = 0.2, *θ* = 0.8, and *λ* = 0.3, the kappa coefficient reached 0.653, which is the maximum value (Table 3), demonstrating that in the ABM model, governmental decision-making behaviors were the most important driving force of the evolution of rural settlements and accounted for 80% of rural settlement changes. In the CA-ABM model, human decision-making behaviors accounted for 70% of rural settlement changes in the transition simulation. Thus, human beings were the dominant decision-making factor in rural settlement changes. Herein, the government played a key role in the changes of rural settlements in the oasis of arid regions due to its macro-control function.

**Table 3:** Multi-scenario simulation in CA-ABM.

	ABM		CA (*λ*)	Kappa coefficient		ABM		CA (*λ*)	Kappa coefficient
	*δ*	*θ*				*δ*	*θ*		
Scenario I	0.5	0.5	0.6	0.355	Scenario III	0.5	0.5	0.4	0.564
	0.65	0.35		0.327		0.65	0.35		0.567
	0.35	0.65		0.364		0.35	0.65		0.592
	0.2	0.8		0.387		0.2	0.8		0.608
	0.8	0.2		0.309		0.8	0.2		0.585
Scenario II	0.5	0.5	0.5	0.404	Scenario IV	0.5	0.5	0.3	0.611
	0.65	0.35		0.418		0.65	0.35		0.623
	0.35	0.65		0.531		0.35	0.65		0.608
	0.2	0.8		0.555		0.2	0.8		0.653
	0.8	0.2		0.449		0.8	0.2		0.602

(Note: Scenario boundary values based on the mean standard deviations of all factors).

We compared the kappa coefficients of 2018 QPT land use results of the PLUS model and the CA-ABM multi-scenario simulation and found that the Markov chain of the PLUS model simulation had a high accuracy. The kappa coefficient was 0.781, which was higher than 0.653 and 0.624 (Table 4). The Markov chain in the PLUS model simulation had an overall accuracy of 0.98.

**Table 4:** Simulation accuracy of 2018 QPT obtained by three methods.

2018		Kappa Coefficients	Overall Accuracy
CA-ABM		0.653	0.87
PLUS	Linear regression	0.624	0.84
	Markov-chain	0.781	0.98

**Fig 8:**
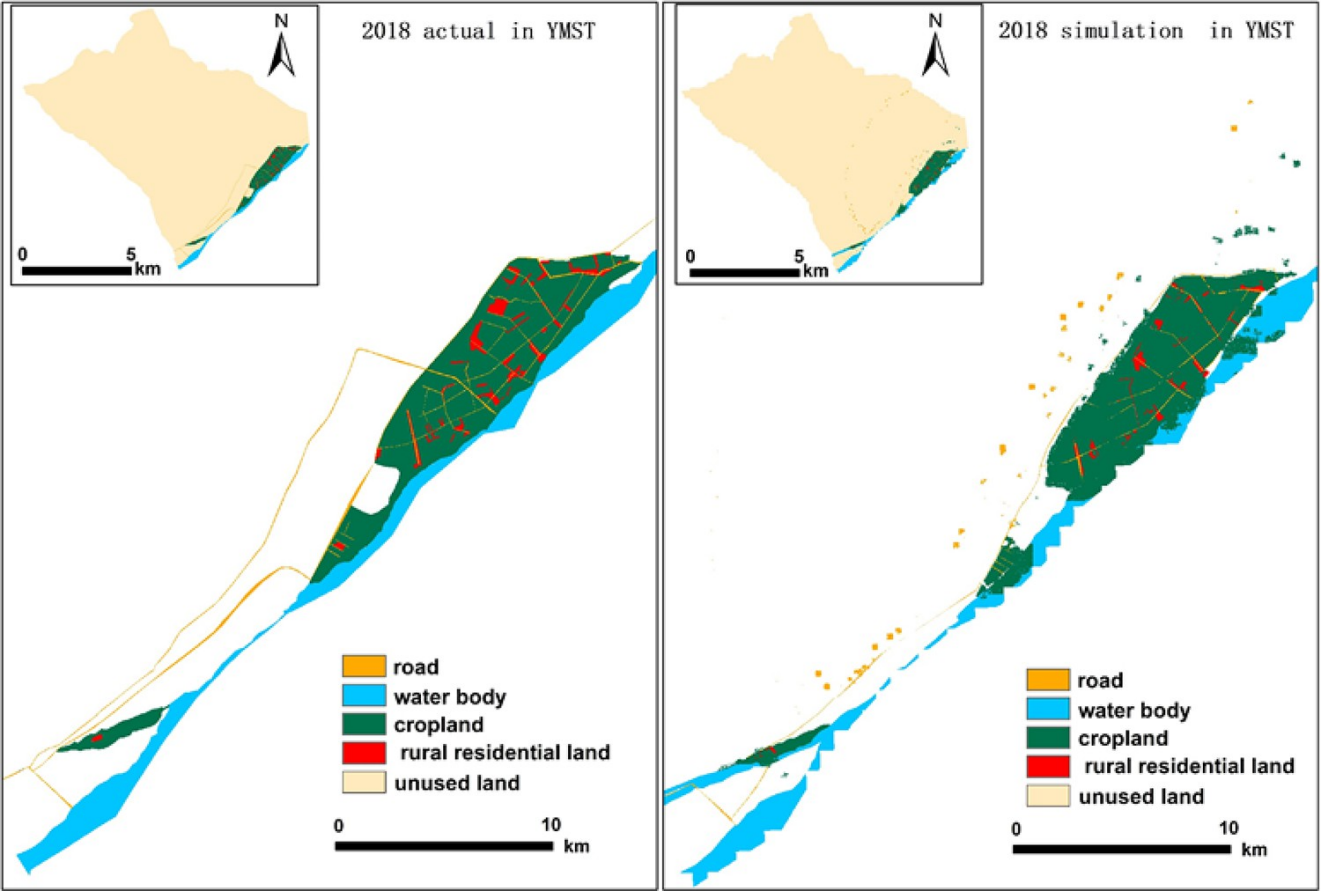
Actual land use in 2018 and simulated land use in 2018 obtained by PLUS of YMST. (Software: ArcMap 10.2.0, http://www.esri.com).

To test the simulation accuracy of the PLUS model, YMST was used as the validation area to simulate the rural settlements and land use types in 2018. The kappa coefficient was 0.731, and the overall classification accuracy was 0.84. However, the simulation accuracy of rural settlements land was relatively low, and the kappa coefficient was 0.709. The simulation results are shown in Fig 8. The result indicated that the Markov chain of the PLUS model could simulate the land use and the evolution of oasis rural settlements in the upper reaches of Tarim Basin.

### Simulation of the evolution of rural settlements in QPT

**Fig 9:**
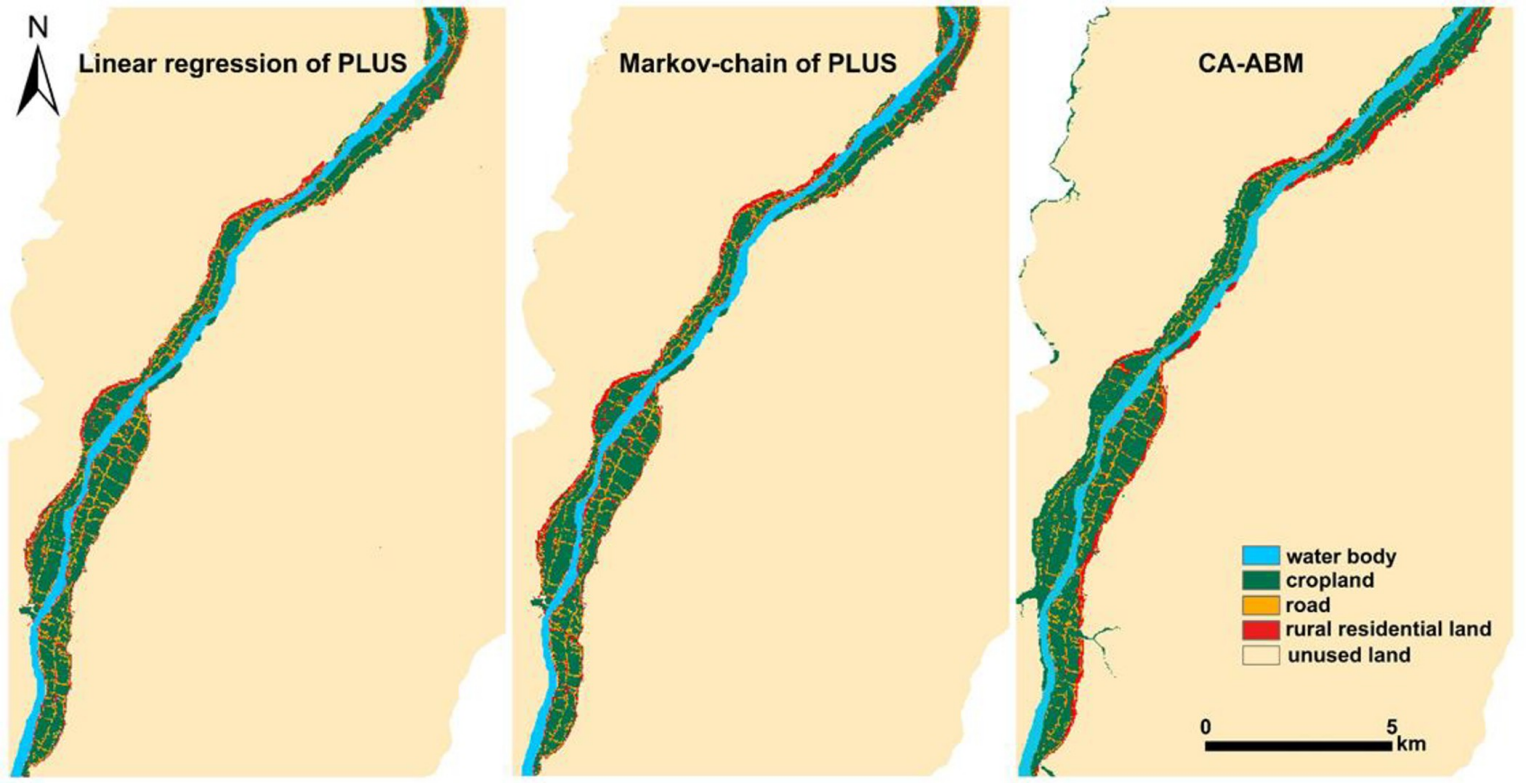
Simulation results of the local spatial distribution of land use in QPT in 2026 obtained by three methods. (Software: ArcMap 10.2.0, http://www.esri.com).

Rural settlements in QPT vary with land use type. The land use in QPT in 2026 simulated by the Markov chain of the PLUS model is shown in Table 5. From the perspective of the time series, the rural residential area increased by a total of 1.3 km^2^ from 2002 to 2010, and it increased by only 0.4 km^2^ from 2010 to 2018. Notably, only a 0.1 km^2^ increase occurred from 2018 to 2026, indicating that the growth rate of QPT rural settlement land will slow down. The cropland, road, and residential area expanded to different degrees, and the area of waters fluctuated and displayed an increasing trend. However, the area of unused land decreased. Rural settlements gradually linearly spread, basically encroaching the cropland and unused land along the road at the edge of the oasis (Fig 9).

**Table 5:** Change in the land use composition of QPT from 2002 to 2026 (km^2^).

Land use types	2002	2010	2018	2026
Cropland	19.6	20.2	20.4	20.7
Unused land	1503.6	1500.8	1499.1	1797.8
Water body	15.1	15.8	16.7	17.2
Road	5.1	5.3	5.5	5.7
Rural residential land	2.8	4.1	4.5	4.6

Table 6 shows the simulation results of the land use in 2026 obtained by three methods. The simulation results of cropland in 2026 showed significant differences. In the CA-ABM simulation results, the cropland area in 2026 was relatively large. The results showed that on the northwest administrative boundary, partial unused land was converted into cropland. In the CA-ABM simulation results, rural residential land in QPT was more concentrated and the original rural residential land was converted into other land use types in the prediction process. The results may not be consistent with the actual situation (Fig 9). In simulation results of the rural residential land of QPT in 2018 obtained with the Markov chain of the PLUS model, the kappa coefficient was 0.896, indicating that the PLUS model could simulate the land use and the evolution of oasis rural settlements in the upper reaches of Tarim Basin. The comparison between the rural residential land use in 2026 and 2018 showed that the original residential land was expanded. The expansion result was consistent with the actual situation.

**Table 6:** Simulation results of land use in 2026 obtained with three methods (km^2^).

2026		Water body	Cropland	Road	Rural settlement	Unused land
CA-ABM		16.5	24.7	5.6	4.8	1494.6
PLUS	Linear regression	16.6	20.5	5.6	4.7	1498.9
	Markov-chain	17.2	20.7	5.7	4.6	1497.8

## Discussion

### Land use conversion in QTP

**Fig 10:**
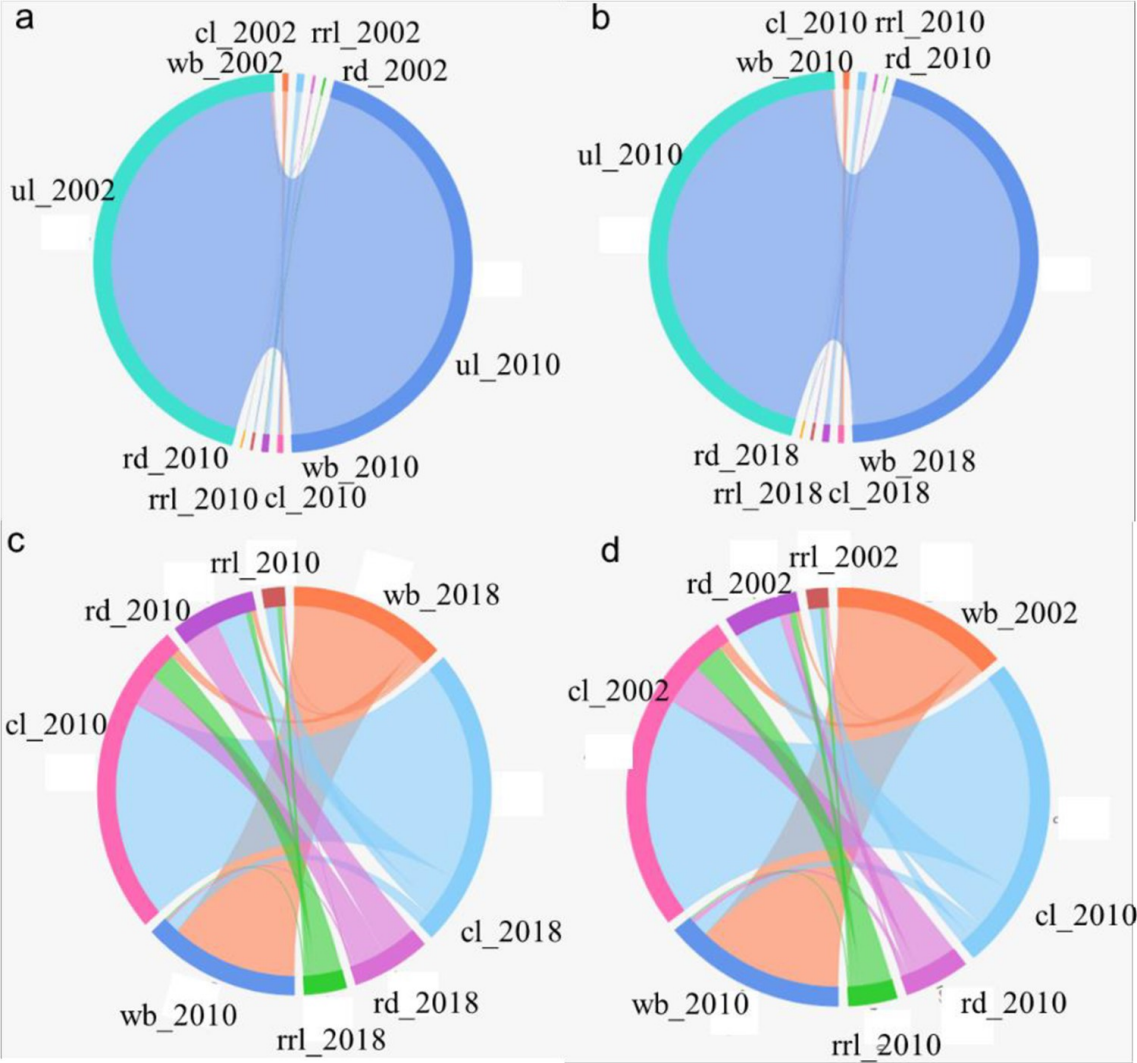
Land use conversion from 2002 to 2018 in QPT. Note: Cropland (cl), rrl (rural residential land), rd (road), ul (unused land), wb (water body).

Unused land accounted for more than 95% of the land area in QPT (Fig 10[A] and 10[B]). Therefore, the conversion among the other four types of land use was not obvious (Fig 10[A] and 10[B]). To highlight the spatiotemporal evolution of rural residential land Fig 10(C) and Fig 10(D) show the chord diagrams of the transfer of water bodies, roads and farmland areas closely related to the temporal and spatial evolution of rural residential land, indicating that the transfer trend of QPT farmland to rural settlements is very obvious. Fig 10(C) presents the changes of the four types of land use from 2010 to 2018. Fig 10(D) shows the changes of the four types of land use from 2002 to 2010. From 2002 to 2010, the increase in rural residential land mainly came from cropland, and the majority of the increase in road area during this period also came from cropland (Fig 10[D]). In the second stage, the conversion of cropland, water body, rural residential land, and road was more intense than in the previous stage, and the total converted area was 3.5 km^2^ (Fig 10[C]). With the increase in the population, rural settlements and cultivated land are expanding, which is brought by the Western Development Strategy. To pursue profits, the area of artificial oases has also gradually expanded from 2002 to 2018. The expansion cannot be unlimited. On the premise of reducing the damage to the ecological environment, the effective arable land area needs to be increased, and engineering technology, biological restoration, and other measures should be taken to improve the quality of land.

### Influencing factors of the evolution of rural settlements in oasis

Oasis evolution in arid areas is influenced by both natural and human factors [14,48–50]. Spatiotemporal variations of rural settlements in the oasis are deeply affected by the natural environment, unlike those in the metropolitan area [12,32]. In the oasis, the industrial and demographic structures are less complex [51]. Therefore, the driving factors selected based on the landscape patch layer could be used to interpret the true changes of rural settlements in the oasis, and the result was consistent with previous studies [37,52]. In addition, water resources significantly affect the development modes of rural settlements in arid regions, including the spatial distribution of rural settlements and the nature of rural life [37]. For example, since two reservoirs were built in the south of Taeragezi Village in the middle reaches of Qipan River, the oasis area in the downstream of the reservoir, including Xieyihela Village, Qiarekelai Village, Tarisi Village, and Qipanyouli Village, has increased significantly. Climate change and hydrological factors also have important effects on the evolution of rural settlements [5,38]. The influences of these two factors on the evolution of rural settlements in the oasis were not discussed because relevant data are not available.

The ABM could solve the uncertainty of human factors in the changes of rural settlements in the oasis of arid regions. This model has two agents—authority agent and resident agent—whose importance varied with time [21]. For instance, in the initial stage of settlement formation, residents’ wishes were the most important in the selection of residential location [37]. With the development of China’s "New Rural Construction" policy, the government began to pay attention to the planning and management of land use in rural settlements, so the governmental decision then became a determinant in the selection of residential location [31]. The coefficients of administrative force indicate that the power of local government in decision-making behavior is indispensable in the process of rural settlement spatial change [53]. This paper demonstrates an approach to analyze and model how farmers’ participation in voluntary mechanisms can affect the landscape structure in rural regions [28]. From the perspective of residents, their household locations showed the feature of familial aggregation, which is also the residential feature of the ethnic minority in the study area. The agglomeration degree of the Uyghur ethnic minority was significantly higher than that of other ethnic minorities [54]. People tend to settle in areas with convenient transportation and education resources and avoid areas with an adverse natural environment such as the Gobi desert and other deserts. In addition, farmers’ income in the study area comes mainly from agriculture. Therefore, convenient agricultural production is also an important factor in the temporal and spatial evolution of rural settlements. Rural settlements are also clustered around administrative centers, indicating that the closer the areas are to grass-rooted administrative centers, the higher the conversion probability of the areas into rural settlements is.

**Fig 11:**
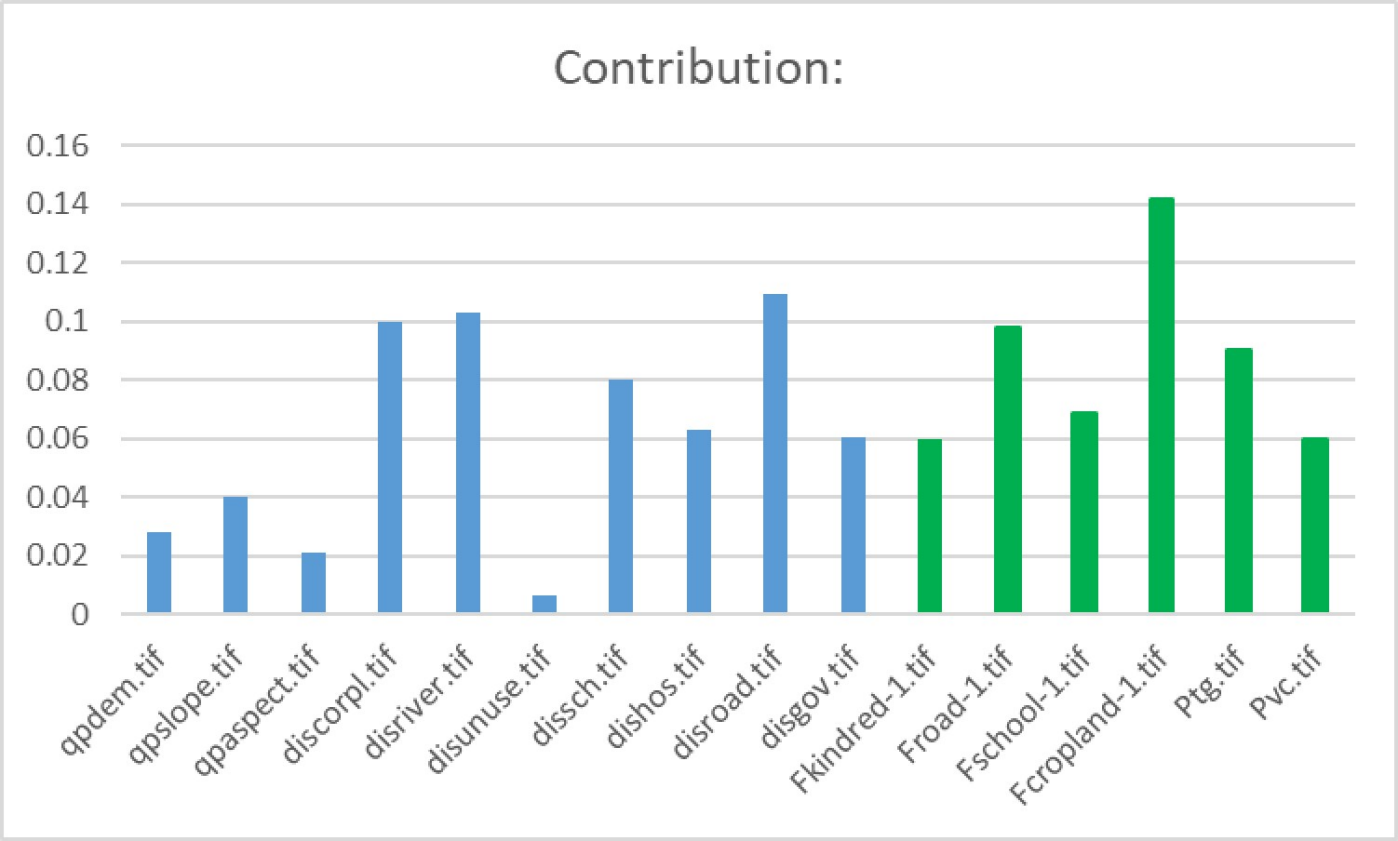
Contribution of each driving factor to the expansion of rural settlement land. (Software: OriginPro 2017C SR2, https://www.originlab.com/).

Moreover, the contribution of all the driving factors to the expansion of each land use type can be exported by the PLUS model. This advantage is important for researchers to understand the underlying causes for land use change. We compared the weights of driving factors for rural settlements in the CA-ABM model and the PLUS model, and found that the weights in the two models were different. First, the natural environmental factors and social economic factors (blue bar chart in Fig 11) were different from the sample survey data (green bar chart in Fig 11). In the CA-ABM model, human decision-making behaviors were the leading factor in the changes of rural settlements in QPT (a weight of 0.7). Second, in the CA model, slope, aspect, water body, and road were the main driving forces for the changes of rural settlements in QPT. However, in the PLUS model, the weights of factors (natural environmental factors, socioeconomic factors, and sample survey data) were calculated by the algorithm in the transfer of rural settlements of QPT. The order of the contributions of these factors to land expansion factors of rural settlements is shown in Fig 11. The contributions of micro-level individual decision-making behaviors and macro-level decision-making behaviors to rural settlement land expansion in QPT are 0.09 and 0.07, respectively. Nevertheless, F_cropland_ is the most important factor that affects the land expansion of rural settlements in the PLUS model. This finding is different from the weights of the influencing factors in the CA-ABM model. In CA-ABM, the weight coefficient is determined by a scenario simulation, but in the PLUS model, the weight depends on the contribution of driving factors. Through multi-scene testing of the CA-ABM model, we found that the management agent made an important contribution to the spatiotemporal evolution of rural settlements in QPT, accounting for 0.56 of the total weight. However, the test of this scenario is based on the preset scenario. We do not know which combination of weight coefficients has the highest simulation accuracy. If other rural oasis settlements in the upper reaches of the Tarim Basin are simulated, then the weight coefficients may be different due to the participation of agents. In this respect, we believe that the PLUS model is superior to CA-ABM. Furthermore, it is worth considering that although natural environmental factors, socioeconomic factors, and sample survey data are used from a different perspective to analyze the transfer of rural settlements in QPT, the overlapping problem of these factors exists, and the overlapping parts may have a positive or negative impact on the simulation results.

This study focused on the changes of rural settlements in the upstream river-side oasis of Tarim Basin with limited water resources in the past 20 years. The governmental macro-control on land use and rural settlements varies with time in reality. The dominant driving factors of oasis changes varied in different periods and interacted with each other. The CA-ABM ignored the parameter variability. In other words, the influences of driving factors on the evolution of rural settlements were assumed to have remained unchanged in different periods.

The driving factors affected by residents’ wishes showed randomness and subjectivity. Hence, the four main factors were confirmed through a field survey, and an improved MCR model was established with composite functions [55]. For instance, the changes of rural settlements could be simulated through an optimal fitting model generated with Gaussian function, trigonometric function, and other functions [29].

### Simulation of the evolution of rural settlements

Even though the accuracy assessment of the change map indicated slight overestimations, the slow annual growth rate of rural settlements (0.045 km^2^) in the study area could be observed from the remote sensing data. The slow growth of rural residential land in recent years was ascribed to the slow growth of the rural population in QPT. Furthermore, most rural settlements expanded at the cost of the reduction of croplands and unused lands. Rural settlements showed an expansion along the main road (Fig 2) due to the fragmentation of croplands [8]. The cropland area increased from 19.6 km^2^ in 2002 to 20.7 km^2^ in 2026. The cropland expanded further due to the development of agriculture and the population growth in oasis. Water from rivers could not meet irrigation demand. and reservoirs have to be constructed to regulate the runoff for irrigation [5,54]. Our results supported the previous finding that oasis expansion, especially cropland expansion, exhibited a significant dependency on water sources [6]. Croplands were mainly distributed along rivers. The critical changes were ignored, such as the increased rural residential land and cropland as well as the decreased unused land, which actually contained important information on environmental changes in the study area [55].

### Spatial distribution of the simulation error

**Fig 12:**
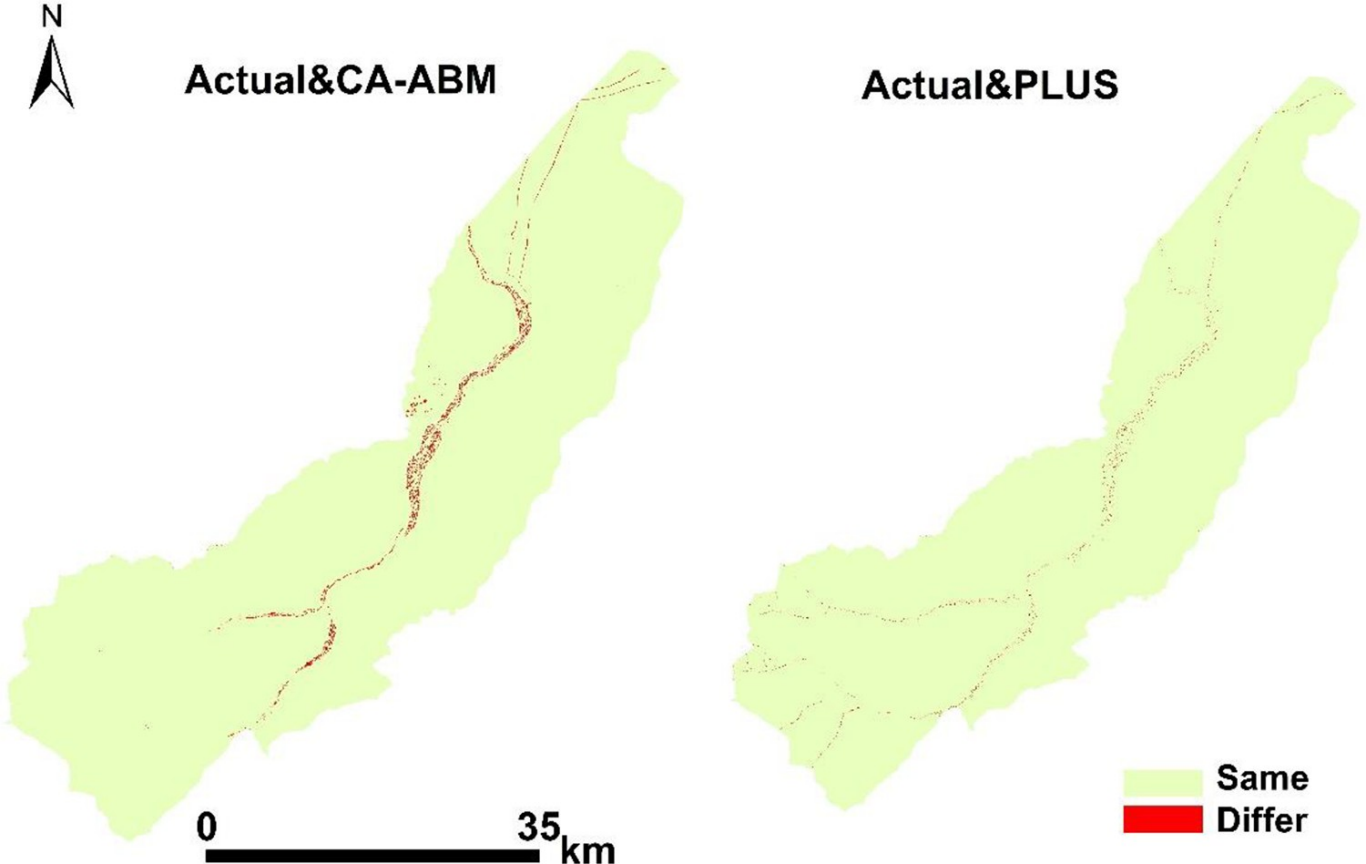
Simulation result and actual map of QPT in 2018. (Software: ArcMap 10.2.0, http://www.esri.com).

On the basis of the Markov chain of the PLUS model and the CA-ABM model, we compared the simulation results of 2018 land use in the QPT area with the actual land use in 2018. The red part in Fig 12 shows the areas where the simulated results are inconsistent with the actual land use results. We found that these errors mainly occurred at the edge of road, rural residential land, and cropland. These errors may be related to the grid scale of land use classification in this study. If more refined land use grid data are used, then the simulation results and actual errors may be reduced.

### Advantages and disadvantagess of the two models

In this study, we compared two models for simulating the spatial pattern of the conversion of rural settlements in the oasis of arid areas. In summary, the Markov chain of the PLUS model is a powerful tool for simulating the conversion of one land use type to rural settlement, and it incorporates the influences of agents and the geographic environment. The Markov chain of the PLUS model was able to accurately simulate the land use types of oases in QPT and YMST, except the land use types of rural settlements. The possible reason for this result is that the pixel of a single rural residential area is less than 30 m, and rural houses and bare land are misclassified. Inevitably, this condition results in the reduced accuracy of the simulation. Second, we consider only some of the major desires and preference conflicts among agents. However, the conflicts and interaction among agents are complex and changeable in reality [9].

A comparison between CA-ABM and PLUS shows that they each have advantages and disadvantages. The combination of ABM and CA equips each fixed cell with the capabilities of social organization and spatial expansion. The ABM-CA model demonstrates the relationship between the geographic environment and agents. However, ABM-CA possesses several limitations for practical applications. We consider only some of the major desires and preference conflicts among agents; the conflicts and interaction among agents are complex and changeable in reality. The simulation results demonstrate the advantages of the PLUS model. However, the limitation associated with this model is that it cannot simulate patch developments, thus causing simulation biases.

## Conclusions

The results of this work showed that remote sensing data could be used to explore the spatiotemporal variation of oasis rural settlements and land use change in Tarim Basin. The PLUS and CA-ABM models were used to simulate the spatiotemporal characteristics of rural settlements affected by the driving factors of land use change in the study area. The Markov chain of the PLUS model was verified in YMST, and the evolution process of oasis rural settlement land and other land use types under the constraints of natural environment was discussed. The leading factors of oasis rural settlement expansion were obtained, and the internal driving mechanism of rural settlement expansion was revealed. The Markov chain of the PLUS model was more accurate than the CA-ABM model in simulating oasis land use types in the upper reaches of Tarim Basin. However, neither of these two models had high accuracy in simulating rural settlement land use in the inner oasis. The development of this region is greatly restricted by water resources. When the regional environment and resource conditions are changed, the simulation accuracy of the model needs to be further verified. Next, we will focus on the spatiotemporal evolution of oasis land use and rural settlements in arid areas under different regional characteristics to expand the application scope of the Markov chain of the PLUS model.
